# The perspective of Canadian health care professionals on abortion service during the COVID-19 pandemic

**DOI:** 10.1093/fampra/cmab083

**Published:** 2021-08-23

**Authors:** Madeleine Ennis, Kate Wahl, Dahn Jeong, Kira Knight, Regina Renner, Sarah Munro, Sheila Dunn, Edith Guilbert, Wendy V Norman

**Affiliations:** 1 Department of Obstetrics and Gynaecology, University of British Columbia, Vancouver, Canada; 2 The School of Population and Public Health, University of British Columbia, Vancouver, Canada; 3 Department of Family Practice, University of British Columbia, Vancouver, Canada; 4 Department of Family and Community Medicine, University of Toronto, Toronto, ON, Canada; 5 Department of Obstetrics and Gynaecology, Laval University, Quebec City, QC, Canada; 6 Department of Public Health, Environments and Society, Faculty of Public Health & Policy, London School of Hygiene & Tropical Medicine, London, UK

**Keywords:** Access to health care, Canada, COVID-19, induced abortion, surveys and questionnaires, transition care

## Abstract

**Background:**

The COVID-19 pandemic and pandemic response created novel challenges for abortion services. Canada was uniquely positioned to transition to telemedicine because internationally common restrictions on abortion medication were removed before the pandemic.

**Objective:**

We sought to characterize the experiences of abortion health care professionals in Canada during the COVID-19 pandemic and the impact of the pandemic response on abortion services.

**Methods:**

We conducted a sequential mixed methods study between July 2020 and January 2021. We invited physicians, nurse practitioners and administrators to participate in a cross-sectional survey containing an open-ended question about the impact of the pandemic response on abortion care. We employed an inductive codebook thematic analysis, which informed the development of a second, primarily quantitative survey.

**Results:**

Our initial survey had 307 respondents and our second had 78. Fifty-three percent were family physicians. Our first survey found respondents considered abortion access essential. We identified three key topicss: *access to abortion care* was often maintained despite pandemic-related challenges (e.g. difficulty obtaining tests, additional costs); *change of practice* to low-touch medication abortion care and *provider perceptions of patient experience*, including shifting demand, telemedicine acceptability and increased rural access. The second survey indicated uptake of telemedicine medication abortion among 89% of participants except in Quebec, where regulations meant procedures were nearly exclusively surgical. Restrictions did not delay care according to 76% of participants.

**Conclusions:**

Canadian health care professionals report their facilities deemed abortion an essential service. Provinces and territories, except Quebec, described a robust pandemic transition to telemedicine to ensure access to services.

**Podcast:**

An accompanying podcast is available in the Supplementary Data, in which the authors Dr Madeleine Ennis and Kate Wahl discuss their research on how family planning care and access to abortion services have changed during the COVID-19 pandemic.

## Lay Summary

Access to abortion care was challenged by the response to COVID-19. Canada had fewer restrictions on medical abortion than many other countries when the pandemic began. The goal of this study was to describe the experiences of health care practitioners providing abortion in Canada and the impact of the pandemic and the pandemic response measures on abortion services. We conducted two surveys of physicians, nurse practitioners and administrators between July 2020 and January 2021. Most of the health care practitioners who participated reported that medical and surgical abortion care were essential and that, except in the province of Quebec, there was a rapid transition to virtual telemedicine care for first trimester abortions. Several practitioners said that virtual care made abortion more accessible. Other practitioners reported that it was challenging to order certain tests, access operating room facilities or make referrals for late second trimester cases. Practitioners felt that patients had strong fears about COVID-19 exposure and reported that limited contraception access was increasingly a reason for seeking abortion care. The results of the study suggested that abortion was considered essential and that the pandemic instigated a transition to virtual care in all provinces and territories except Quebec.

**Key Messages**

- Access to abortion care could potentially be jeopardized by the COVID-19 response.
- Unique regulations enabled Canada to deliver virtual abortion through primary care.
- Evidence-based virtual abortion services may increase access to care.
- Regulatory factors influence jurisdictions’ abilities to deliver care.

## Background

Access to essential, safe and comprehensive abortion care was jeopardized by the global response to the COVID-19 pandemic in early 2020. Restrictive measures designed to limit the spread of the virus, including those on nonessential travel and medical care, affected access to reproductive and sexual health care (1). Legal and regulatory responses to this challenge were often regressive in international jurisdictions where abortion access was already limited (2–5). In other jurisdictions, progressive responses focussed on preserving access through telemedicine for early medical abortion (MA) care, since this minimized clinical points of contact and decreased the risk of COVID-19 exposure. For example, the UK, France, Australia and New Zealand temporarily allowed telemedicine for early MA, removed requirements for routine screening ultrasounds and laboratory testing and increased gestational age limits for early MA to 10–13 weeks of gestation (6–8).

Canada was uniquely positioned to transform MA care insofar as several of these requirements regarding MA (mandatory screening ultrasound, physician dispensing of mifepristone) were removed prior to the pandemic (9–11). The Society of Obstetricians and Gynaecologists of Canada (SOGC) also clearly stated at the beginning of the pandemic that abortion care is an essential service, and access needed to be maintained (12,13). Abortion care is governed in Canada in the same way as any other reproductive health service, and is not regulated by criminal law. The decision to have an abortion is solely between a pregnant person and their health care provider and is not restricted by indication (e.g. foetal anomaly) or gestational age (9). By 2020, many primary care providers in Canada had incorporated first trimester MA into their clinical practices. An exception exists in the province of Quebec, which uniquely maintained restrictions that effectively limited MA care, and promoted surgical services (14,15). Barriers to providing MA care in Quebec include restrictive provincial medical licensing body policies, restrictive facility approaches with perceived vested interests in preserving surgical provision, lack of inter-professional support and general professional uncertainty about the regulations (14).

A growing body of evidence points to the safety and acceptability of low- or no-touch telemedicine abortion. An analysis of more than 52 000 MA in the UK showed that the telemedicine‐hybrid model for care adopted in response to the pandemic was as effective, safe, acceptable and more accessible than conventional care provided in the first 3 months of 2020 (16). These findings align with other research showing that home-based and telemedicine first trimester MA are safe with high rates of efficacy and acceptability (17,18). Existing research in several high-income nations also suggests providers may be interested in maintaining the liberalized practice changes implemented since the beginning of the COVID-19 pandemic (19,20). However, more research is required to assess the sustainability of these regulatory changes. Turning to Canada, the impact of the COVID-19 pandemic and pandemic conditions on abortion care is unknown.

Therefore, our objective was to characterize the experiences of health care practitioners on the impact of COVID-19 and pandemic response measures on abortion care in Canada, with a focus on access, telemedicine and early MA provision.

## Methods

We conducted an exploratory sequential mixed methods study that involved the collection and analysis of qualitative data from our 2019 Canadian Abortion Provider Survey (21) which, in turn, informed the development of a second primarily quantitative survey. We adapted the Standards for Reporting Qualitative Research Checklist for a mixed-methods approach (22). We conducted this second survey to further understand and quantify factors identified in the first survey through a refined set of questions and in response to a request from federal regulatory stakeholders.

### Survey 1: The Canadian Abortion Provider Survey

#### Data collection

Between July and December 2020, we conducted a self-administered, anonymized, cross-sectional survey of Canadian physicians, nurse practitioners and administrators who provided first, second or third trimester medical or surgical abortion provision in 2019 (21). The University of British Columbia Children’s and Women’s Hospital Research Ethics Board approved the survey (UBC-CW REB, H18-03303). It was available in English and French on the Research Electronic Data Capture (REDCap) platform, and included a consent form, as well as sections on demographics, clinical characteristics of abortion care and stigma experienced by providers. We distributed the survey through health care professional networks, using a modified Dillman technique to maximize participation (23). We included a non-mandatory, open-ended question: ‘What impacts has Covid-19 had on your individual abortion practice and/or access to abortion in your province?’

#### Data analysis

ME, KW and KK conducted a codebook thematic analysis (24) of open-ended responses following an inductive approach. We each read the same 50 responses to familiarize ourselves with the data and then independently coded these responses. Next, we compared our analyses, and agreed on an initial set of codes and descriptions for the codebook. We then divided the full set of responses and coded these independently. Subsequently, we refined existing codes and descriptions and agreed on new codes that were identified in the independent analysis. We used the revised codebook in a final analysis of the data, after which we organized the codes into topics related to the research question (see Supplementary Data). In the context of this low-inference approach, contemporaneous team discussions helped identify how personal attributes, qualifications and assumptions interacted with the data and analysis.

### Survey 2: Community of Practice Survey

We used the identified topics in the Survey 1 analysis to develop a second survey to assess the impact of the pandemic on the provision of Canadian abortion services. From December 2020 to January 2021, we conducted the survey (UBC-CW REB, H16-01006), which included a consent form and questions assessing brief demographics, monthly MA volume from January to September 2020, previous experience providing MA via telemedicine, and the impact of the COVID-19 pandemic on the provision of abortion services (see Supplementary Data). We excluded respondents practicing in Quebec from analysis of MA-related questions, as restrictive medical policies sustained providers’ preference for surgical abortion and provincial/administrative inertia limit access to MA (15,25). This survey was available in English and French and we invited members of the *Canadian Abortion Providers Support—Communauté de pratique canadienne sur l’avortement* network (a national community of practice to support mifepristone abortion practice) via registered email (10,26). As Survey 1 also recruited through this platform, there may have been overlap in participants. We generated summary statistics on R, version 3.6.1 and applied the Survey 1 codebook to open-ended responses.

## Results

### Survey 1 results

A total of 307 participants responded to the pandemic-related open-ended question. Their demographics are provided in Table 1. All participants confirmed they completed the survey only once, were no longer in training, and independently provided abortion care in Canada. Twelve participants indicated they were both clinicians and administrators.

**Table 1.** Demographics of respondents to the COVID-19 open-ended question in the Canadian Abortion Provider Survey conducted in 2020 (*n* = 307)

Province^a^, *n*	
British Columbia	66
Alberta	13
Saskatchewan	7
Manitoba	8
Ontario	93
Quebec	73
Prince Edward Island and Newfoundland and Labrador	7
New Brunswick	11
Nova Scotia	18
Territories	11
Role, *n*	
Clinicians	280
Administrator	39
Profession^b^, *n*	
Physician	262
Nurse practitioner	18
Specialty^c^, *n*	
Family physician	163
General OB/GYN	69
OB/GYN with MFM subspecialization	25
Other	5
Provision, *n*	
First trimester MA	212
First trimester surgical abortion	114
Second trimester surgical abortion	55
Second trimester MA	55
Third trimester MA	35
Age (median, range)	41 (26–76)
Gender, *n*	
Women	234
Men	46
Other	0

MFM, maternal–foetal medicine; OB/GYN, obstetrician–gynaecologists.

^a^Some provinces and territories were combined for confidentiality purposes.

^b^Profession of clinicians; does not include administrators.

^c^Speciality of physicians; does not include administrators or nurse practitioners.

We identified three common topics related to the impact of the COVID-19 pandemic on abortion care in Canada: access to care, change in practice and perceptions of the patient experience.

#### Access to abortion care

Many abortion providers indicated that the pandemic did not affect their ability to provide access because they or their province considered abortion care essential. One family physician from Alberta (ID 110) said, ‘In our community, we have considered abortion and contraceptive care to be an essential service and have made sure access continued throughout COVID restrictions’. Several participants described an increase in access to care during the pandemic, ‘We have found that many patients throughout the province have utilized our service due to an increase in accessibility that accompanies telemedicine, and we are hopeful to continue to offer this care. However, we are also aware that for patients who do not have access to a phone/internet, telemedicine is not accessible’ (administrator, Ontario, ID 27). Some participants providing in-person care noted that reduced clinic hours had affected access. Barriers to timely care included challenges accessing tests, for example ‘Difficulty getting ultrasounds, labs done (less staff for both for booking/performing) [and] difficulty communicating with other offices as people are generally more busy and understaffed’ (administrator, British Columbia, ID 272). Some abortion providers reported fewer requests for abortion (both from patients and through referrals) and a number of surgical providers indicated that access to operating theatres was limited. Finally, several participants perceived that their patients had experienced limited access to contraception care.

#### Change of practice

The majority of respondents reported a change from in clinic to low- or no-touch care with virtual components. The degree of this change varied from ‘I have shifted the first or subsequent visits to telephone visits but usually require in person assessment at least once’ (family physician, British Columbia, ID 6) to ‘I have offered entirely telehealth abortion care for early first trimester pregnancies. Minimized investigations—bloodwork initially and clinically history of heavy bleed and resumption of menses as confirmation’ (family physician, British Columbia, ID 196). Some first trimester MA providers prescribed a second dose of misoprostol routinely. Others indicated increasing gestational age limits for second trimester in hospital abortions/labour inductions when patients could not be referred elsewhere. An obstetrician–gynaecologist (British Columbia, ID 164) described, ‘We are providing medical abortion care to women ≥25 weeks gestation with fetal anomalies/genetic anomalies that we would have normally referred to a US centre’.

Many participants described positive experiences, for example ‘We have moved quite seamlessly to no-touch medical abortion services and this has been quite successful and rewarding’ (nurse practitioner, Ontario, ID 498). Participants who did experience difficulties with the transition to low- or no-touch care identified resource issues, including increased costs to adhere to infection prevention and control measures as well as staffing shortages resulting from secondment to other roles, new childcare demands or limited ability to travel between clinics. Depending on jurisdiction, billing contributed to or mitigated costs. In Alberta, a family physician (ID 29) described ‘the telephone visit code really doesn’t compensate for the length of time spent counselling pre and post abortion’ whereas in Nova Scotia, a family physician (ID 284) highlighted ‘Provincial fee to provide medical care via telephone has been helpful’.

#### Perceptions of the patient experience

Many respondents commented on patient behaviour and experiences. A common observation was that demand for care decreased in the early months of the pandemic, and providers hypothesized that this was due to decreased frequency of intercourse as well as fears about contracting COVID-19, as a family physician from the Territories (ID 691) described, ‘Patients are more reluctant to travel out of territory to access abortion care beyond the first trimester because they are terrified of the virus, and do not want to self-isolate at hotel hubs for 14 days before re-entering the territory’. Participants shared anecdotes about patients who presented for care at a later gestational age or continued with pregnancy because of fears about contracting COVID-19 or, in one case, because a patient tested positive for the virus and was unable to travel for care. Participants generally reported that patients were more likely to request MA care over surgical, felt that patients were anxious, and noted that at most clinics, support people were not permitted to attend appointments.

### Survey 2 results

The survey was completed by 78 respondents. Their demographics are provided in Table 2. The number of MAs provided monthly by respondents increased slightly in April 2020, with the mean volume ranging from 4.9 to 5.8 prior to April 2020, and ranging from 6.1 to 7.3 after April 2020. Since March 2020, 91% of respondents had provided medical or surgical abortion services. We did not identify any new codes in the qualitative analysis of the open-ended questions.

**Table 2.** Demographics of the Community of Practice Survey respondents (2020–21; *n* = 78)

Province^a^, *n*	
Western Provinces^b^	32
Ontario	29
Quebec	10
Maritime Provinces and Territories^c^	7
Role, *n*	
Physicians	65
Nurse Practitioners	6
Pharmacists and Administrators	7
Geography, *n*	
Urban	51
Rural	25

^a^Some provinces and territories were combined for confidentiality purposes.

^b^Included participants from British Columbia, Alberta, Saskatchewan and Manitoba.

^c^Included participants from New Brunswick, Newfoundland and Labrador, Nova Scotia, Northwest Territories and Yukon.

#### Volume

Among respondents who reported providing MA during the pandemic (*n* = 61), 52.5% reported that the number of MAs increased, 41% reported that the number of MAs did not change and 6.5% reported a decrease in the number of MA.

#### Timely access

Among respondents who have provided medical and/or surgical abortion during the pandemic (*n* = 63), 76% reported that their patients did not experience delays in care due to restrictive measures.

#### Telemedicine

Among respondents who have provided first trimester MA virtually (*n* = 55), 83.6% had no experience providing MA virtually before the pandemic. However, 88.9% responded that they had provided MA virtually since the pandemic. The majority of respondents providing first trimester MA virtually offered pre-abortion consultation/counselling, prescription and follow-up virtually (90.9%, 85.5% and 90.9%, respectively). Forty-nine percent responded that they provided virtual emergency care.

Furthermore, respondents who provided first trimester MA virtually during the pandemic (*n* = 48) were asked about situations where they required testing, summarized in Table 3.

**Table 3.** Requirements for clinical tests by Canadian medical abortion providers during the COVID-19 pandemic (2020–21; *n* = 48)^a^

	Always, *n* (%)	As indicated, *n* (%)	Never, *n* (%)	Not applicable, *n* (%)
Ultrasound	9 (18.8)	39 (81.2)	0 (0)	0 (0)
Rh testing	17 (35.4)	20 (41.7)	10 (20.8)	<5
Serum bHCG	28 (59.6)	18 (38.3)	<5	0 (0)
Haemoglobin	25 (55.6)	16 (35.6)	<5	0 (0)
STI testing	18 (37.5)	25 (52.1)	5 (10.4)	0 (0)

STI, sexually transmitted infections. <5 is used to ensure confidentiality of participants.

^a^Does not include participants from Quebec; only includes participants who provided first trimester medical abortion care during the COVID-19 pandemic.

Finally, respondents who have provided medical and/or surgical abortion during the pandemic (*n* = 63) were asked about difficulties mentioned by patients in accessing abortion services, summarized in Table 4. The most common mentioned difficulty was fear of exposure to COVID-19 in public locations.

**Table 4.** Difficulties for patients to access services during the COVID-19 pandemic (2020–21) as reported by Canadian abortion providers (*n* = 63)

	Number of providers who responded that their patients mentioned experiencing difficulty, *n* (%)
Difficulty obtaining information on abortion services	16 (25.4)
Delay in getting a referral for abortion care	16 (25.4)
Social distancing	8 (12.7)
Lockdowns or decrease transportation between regions	12 (19.0)
Interruption of local transportation	11 (17.5)
Increase of restrictive legal measures against abortion	0 (0)
Re-organization of medical services	11 (17.5)
Medical leaves of health professionals	<5
Change in opening/closing hours of shops, clinics or community organizations	13 (20.6)
Fear of getting COVID in public locations	28 (44.4)
Being diagnosed with a positive test for COVID-19	9 (14.3)
Lack of privacy for medical abortion at home	<5
Intimate partner violence	10 (15.9)
Loss of income	14 (22.2)
Difficulty related to technology	15 (23.8)

<5 is used to ensure confidentiality of participants.

## Discussion

This study, which assesses changes in access to Canadian abortion services since the COVID-19 pandemic, is novel, timely, and has the potential for rapid impact on policy. Most abortion providers in most Canadian provinces and territories reported a seamless switch to providing a higher proportion of MA compared with surgical abortion, and an increase in telemedicine. Though several difficulties were reported most strikingly in Quebec, this transition was enabled by the support and pandemic guidelines of SOGC (12).

Our findings indicate that the availability of abortion care was maintained and a rapid transition to telemedicine MA was experienced, in provinces and territories except for Quebec. This contrasts to the experiences reported in some jurisdictions internationally (1,4,5,19,27–29). For example, a rapid response survey of independent abortion clinics in the USA showed that, 51% of clinics, clinicians or staff had been unable to work because of the pandemic or public health response (27). Across many European nations, the USA and in other jurisdictions (3,5), legal and regulatory restrictions on abortion care have been a barrier to following evidence-based guidelines for pandemic abortion care (2,27). In contrast, the Canadian transition to pandemic abortion care, similar to that reported by Gibelin in France (19) and Aiken in the UK (16), was quickly implemented and deemed essential. Rapid transition to telemedicine first trimester MA was likely facilitated by the removal of restrictions by the federal drug regulator Health Canada in 2019, and hindered by the ongoing restrictive regulations in Quebec (14,15,30). The supportive regulatory framework—which did not prescribe testing—enabled providers to adopt evidence-based guidelines for abortion care during pandemics and periods of social disruption beginning in April 2020 (12,13), including reductions in the indications to order pre-procedural ultrasound, bHCG and Rh factor tests.

The results of this study may have important implications in jurisdictions seeking to advance reproductive health during and after the COVID-19 pandemic response. Our findings suggest that low- or no-touch MA provided in primary care may increase geographic equity in access to safe care. This approach has the potential to address barriers to in-clinic care, such as the burden among patients who care for others. Our results indicate that consideration of patient economic status (results of Survey 2) and access to communication technology (results of Surveys 1 and 2) may be important for optimizing equitable access to abortion. Finally, a comprehensive approach to reproductive health care is vital, since considering contraception care as nonessential or implementing policy restricting dispensation (31), may have negative consequences even where access to abortion care is preserved.

The experiences of health professionals providing abortion care in Canada during the COVID-19 pandemic illustrates opportunities for evidence-based provision of low- and no-touch first trimester MA in primary care and providing advanced gestational age abortions closer to the patient’s home rather than referring them elsewhere including to the USA. Strengths of the study include the large national sample and the integration of qualitative and quantitative data, which provided a rich contextual understanding of the Canadian transition to pandemic abortion care from a front-line perspective. Interpretation of the results may be limited by the potential for overlap in participants between the surveys. A further limitation is that the patient experience of pandemic abortion care is described from the perspective of health care professionals. The second survey had a smaller sample size than the first. We sought understanding to assist interpretation of our qualitative findings and thus fielded the quantitative questions using a limited recruitment method through one network, that was open to respondents for 2 months. While beyond the scope of this study, future research led by our team will investigate patient experiences, including perceptions of telemedicine abortion care and safety of low- and no-touch abortion care in Canada. Further investigation into provincial/territorial differences in care and access, including factors explaining the persistence in restrictive policies on first trimester MA care in Quebec, would allow for more targeted knowledge translation and policy development plans.

In the 10 months following the outbreak of the COVID-19 pandemic, Canadian abortion providers from all provinces except Quebec, reported quickly and easily transitioning to the provision of virtual MA. This transition was influenced by previous federal removal of regulatory restrictions, and by the rapidly developed and disseminated national guidelines on virtual abortion care. Our results highlight opportunities to optimize equitable abortion care both in Canada and internationally.

## Supplementary Material

cmab083_suppl_Supplementary_File_1

## Data Availability

Our ethics approval has specified the primary data are not available.

## Declaration

Funding: both surveys were approved by UBC Children’s and Women’s Research Ethics Board (H18-03303 and H16-01006). Survey 1 (The Canadian Abortion Provider Survey) was funded by The Canadian Institutes of Health Research (CIHR) and Survey 2 by CIHR (PHE148161, CPP-329455-107837) and by The Michael Smith Foundation for Health Research (Award #16743). Dr Norman is supported by a CIHR and Public Health Agency of Canada Chair in Applied Public Health Research (2014–24, CPP-329455-107837). In kind support was provided by the Women’s Health Research Institute of the British Columbia Women’s Hospital and Health Centre of the Provincial Health Services Authority of British Columbia. The SOGC supported development and design of the Community of Practice Platform, through a contract with the corresponding author’s institution.

Ethical approval: none.
